# Economic evaluation of upper limb prostheses in the Netherlands including the cost-effectiveness of multi-grip versus standard myoelectric hand prostheses

**DOI:** 10.1080/09638288.2022.2151653

**Published:** 2022-12-19

**Authors:** Nienke Kerver, Elise Karssies, Paul F. M. Krabbe, Corry K. van der Sluis, Henk Groen

**Affiliations:** a Department of Rehabilitation Medicine, University of Groningen, University Medical Center Groningen, Groningen, The Netherlands; b Department of Epidemiology, University of Groningen, University Medical Center Groningen, Groningen, The Netherlands

**Keywords:** Upper extremity, prostheses, amputation, artificial limbs, questionnaire, costs and cost analysis, quality of life, patient reported outcome measures

## Abstract

**Purpose:**

To investigate the costs, quality of life, and user experiences associated with upper limb prosthesis use, and to evaluate the cost-effectiveness of multi-grip compared to standard myoelectric hand prostheses (MHPs/SHPs).

**Materials and methods:**

The EQ-5D-5L to assess the quality of life, the patient-reported outcome measure to assess the preferred usage features of upper limb prosthesis (PUF-ULP), and a cost questionnaire (societal perspective) were completed by 242 prosthesis users (57% men; mean age = 58 years). Incremental cost-utility and cost-effectiveness ratios (ICUR/ICER) with respectively the EQ-5D-5L and PUF-ULP were calculated to compare MHPs with SHPs. Statistical uncertainty was estimated using bootstrapping. Netherlands Trial Registry number: NL7682.

**Results:**

The mean yearly total costs related to prosthesis use of MHPs (€54 112) and SHPs (€23 501) were higher compared to prostheses with tools/accessories (€11 977), body-powered (€11 298), and cosmetic/passive prostheses (€10 132). EQ-5D-5L and PUF-ULP scores did not differ between prosthesis types. ICUR was €-728 833 per quality-adjusted life year; ICER was €-187 798 per PUF-ULP point gained.

**Conclusions:**

Myoelectric prostheses, especially MHPs, were most expensive compared to other prostheses, while no differences in quality of life and user experiences were apparent. MHPs were not cost-effective compared to SHPs. When prescribing MHPs, careful consideration of advantages over SHPs is recommended.

## Implications for Rehabilitation

- Myoelectric upper limb prostheses, especially the multi-grip hands, were more expensive than all other types of upper limb prostheses.
- Health-related quality of life and user experiences were comparable in users of different types of upper limb prostheses.
- Acquisition costs mainly explained the differences in costs related to upper limb prosthesis use.
- Prescription of multi-grip hand prostheses should be considered carefully, since these are not cost-effective compared to standard myoelectric hand prostheses.

## Introduction

Upper limb absence can affect an individual’s life tremendously. Literature reports lower health-related quality of life among people with congenital upper limb absence compared to the general population [1]. Additionally, lower life satisfaction was found in people with acquired upper limb amputation compared to the general population [2]. An upper limb prosthesis may offer a functional or aesthetic solution for individuals with upper limb absence. Furthermore, higher health-related quality of life [3,4] and employment rates [3,5] were reported among upper limb prosthesis users compared to non-users, which underline the potential benefits of upper limb prostheses.

Although the total average cost of upper limb prosthesis related healthcare in the Netherlands per user increased by 17% from €4160.- in 2016 to €4850.- in 2020 [6], doubts exist whether these extra costs have been used effectively. The increased cost may be attributed to the more frequent prescription of expensive multi-grip myoelectric hand prostheses (MHPs), such as the Bebionic (Ottobock; Duderstadt, Germany), Vincent (Vincent Systems; Weingarten, Germany), or i-Limb (Touch Bionics; Livingston, UK). The latter was the first MHP introduced on the market about 15 years ago. An MHP is controlled with electrical signals generated by the muscles of the remnant limb, has five moveable fingers, can perform multiple grips, such as the tripod grip, power grip, pinch grip, and pointing the index finger [7,8]. In comparison, standard mono-grip myoelectric hand prostheses (SHPs), such as the Myohand Variplus Speed (Ottobock; Duderstadt, Germany) or Motion Control Hand (Fillauer; Salt Lake City, USA), can only perform a tripod grip, although they are controlled in the same way as MHPs [8]. A further illustration of the increased costs of upper limb prostheses is provided by Blough et al. [9], spanning a period of several decades. They estimated that 5-year costs of prosthetic and assistive devices increased by 277% from $31 129 for Vietnam War veterans around the 1970s to $117 440 for Operation Iraqi Freedom/Operation Enduring Freedom veterans in the early 21st century [9]. The Operation Iraqi Freedom/Operation Enduring Freedom group not only used a prosthesis relatively more often, but also used more advanced types of prostheses compared to the Vietnam group [9]. However, the provision of the advanced, high-cost MHPs has not been supported by health economic evaluations, an evidence gap which is signalled in multiple reviews [10–12]. Remarkably, these reviews also could not identify health economic evaluations regarding other types of upper limb prostheses, such as the SHPs, body-powered prostheses (BPs), cosmetic/passive prostheses (CPs), and prostheses with tools/accessories [10–12].

Potentially, there is a lot to gain for policymakers, health insurance companies, clinicians, and prosthesis users themselves from a better understanding of the costs, effects, and the cost-effectiveness of upper limb prostheses. Important in this respect is that 4–50% of the people with upper limb absence seems to reject their upper limb prosthesis [3,13–15]. The variety in rates of prosthesis rejection between studies may be explained by differences between the included populations, such as the level of upper limb absence, inclusion of people with bilateral upper limb absence, country in which the study was performed, and method of patient recruitment [3,13–15]. A recent study regarding upper limb prosthesis abandonment, which included 25 Austrian respondents with traumatic upper limb injury, found no significant difference in prosthesis acceptance rates between respondents who were amputated before or after 2006 despite the availability of MHPs in the latter period [13]. These results further increase the doubts about the cost-effectiveness and user experiences of upper limb prostheses, especially regarding the costly MHPs. Additionally, studies that compared the MHP and SHP provided mixed claims about the advantages of MHPs over SHPs. For instance, one study found better dexterity with the MHP compared to the SHP [16], while three other studies did not find better dexterity or prosthetic hand function in MHPs [17–19]. Consequently, the benefits of the MHP in comparison with the SHP are still unclear, particularly when related to the higher costs of the former. This study aimed to provide a broad overview of the costs, health-related quality of life, and user experiences associated with the use of a variety of prosthesis types in adult prosthesis users (age ≥18 years) with major unilateral upper limb absence. Secondly, we aimed to evaluate the cost-effectiveness of the MHP compared to the SHP for adult prosthesis users.

## Material and methods

We conducted and reported our health economic evaluation according to the Dutch guidelines [20] and the consolidated health economic evaluation reporting standards statement [21]. A societal perspective was adopted, which entails all costs and consequences regardless by whom these were incurred [20,21]. For this study, individual patient-level data were gathered in a nationwide survey. Individual costs and effects are presented over a time horizon of one year. The local Medical Ethics Review Board of the University Medical Centre Groningen waived formal study approval (METc 2018/582). This study was carried out in compliance with the Declaration of Helsinki. Participants were asked to sign an informed consent before completing the survey. This study is registered in the Netherlands Trial Registry: NL7682.

### Survey development

The survey consisted of questions regarding patient demographics (i.e., age, sex, side of upper limb absence, origin of upper limb absence, prosthesis type, prosthesis experience, prosthesis wearing time, educational level, job), and three other parts: 1) health-related quality of life, 2) patient-reported outcome measure to assess the preferred usage features of upper limb prosthesis (PUF-ULP) [22], and 3) costs related to upper limb prosthesis use.

#### Health-related quality of life

Health-related quality of life was measured with the Dutch version of the EQ-5D-5L [23,24]. This instrument comprises five questions (mobility, self-care, usual activities, pain, anxiety/depression) each with five response levels (no problems, slight problems, moderate problems, severe problems, and extreme problems/unable to do something) [23,24]. A unique health state is defined by combining one level from each of the five questions. Subsequently, the Dutch scoring algorithm for the EQ-5D-5L was applied to generate a single value that expresses the health status of an individual respondent [25]. The values range from −0.446 to 1, with higher scores indicating better health-related quality of life [25]. The EQ-5D-5L also contains a visual analogue scale (VAS) on which participants were asked to rate their perceived health on a scale ranging from 0 to 100, again with higher scores indicating better perceived health. Although the EQ-5D was developed as a generic instrument to measure health-related quality of life and investigation of measurement properties were rare, results of a systematic literature review suggest good reliability and validity of the EQ-5D in people with upper extremity conditions other than upper limb absence [26].

#### PUF-ULP

A more specific outcome measure regarding body-worn devices was used in addition to the health-related quality of life: the PUF-ULP. The PUF-ULP is an electronic patient-reported outcome measure that runs in the HealthSnApp application (www.chateau-sante.com/healthsnapp). This is a flexible tool, with interactive routines; it runs on smartphones and computers, and is highly configurable from a web-based console module. The content of the PUF-ULP was developed specifically for this study as previously described [22] and was designed to reflect the extent to which an individual’s prosthesis meets the preferred usage features of upper limb prostheses [22]. The included items of the PUF-ULP were identified by 358 Dutch individuals with upper limb absence, largely corresponding to the target population of the current study [20]. This resulted in nine items: “wearing comfort,” “functionality,” “independence,” “work, hobby, and household,” “user-friendliness,” “life-like appearance,” “phantom limb pain,” “overuse complaints,” and “reliability.” The measurement model combines elements of item-response theory and the Rasch model [27–29]. The underlying framework for this method has previously been applied to other study populations [30,31].

The PUF-ULP measurement consisted of two tasks. In the first task, participants were asked to rate their experiences with their upper limb prosthesis based on the nine items (Supplementary file 1) [22]. Each item has four response levels (e.g., comfortable, fairly comfortable, not very comfortable, uncomfortable). In the second task, six slightly modified (hypothetical) descriptions of Task 1 were presented. Respondent were asked to indicate whether their experiences with their own prosthesis were better or worse than the hypothetical descriptions [22]. The hypothetical user experiences were constructed in such a way that one item was better than the respondent’s own experience and one item was worse. Based on the responses, weights were estimated for each level of each of the items [29]. Subsequently, a single score was calculated by adding up all weights. The lowest and highest possible scores, if each item was rated on respectively the worst or best level, were −12.0 and 0.1. However, these raw scores were transformed, by adding up 12, to scores ranging from 0 to 12.1 for ease of interpretation of the health economic evaluation results. Higher scores indicate that the prosthesis better meets the preferred usage features of upper limb prostheses.

#### Costs related to upper limb prosthesis use

To determine the costs related to upper limb prosthesis use, the Productivity Cost Questionnaire [32] and the Medical Consumption Questionnaire [33] were adjusted to the situation of Dutch upper limb prosthesis users. Although no validity studies were performed yet, most questions of the Productivity Cost Questionnaire, except for the module “productivity losses related to unpaid work,” were derived from existing validated questionnaires [34]. Furthermore, the Medical Consumption Questionnaire was developed in the Netherlands, and as such tailored to the healthcare organization in the Netherlands [33]. Direct medical costs (e.g., the cost of acquisition and repairs, homecare, and outpatient visits), informal care, and travel expenses (i.e., expenses for travelling related to prosthetic care, such as visits to prosthetist, therapist, or doctor) were derived from the questionnaires. Questions about appointments with a speech therapist and dietician were replaced by questions about appointments with a hand therapist, prosthetist, and a technician producing adaptive devices or prosthetic accessories. Furthermore, the questions regarding emergency room visits and ambulance transport were removed, while a question about visits to the rehabilitation centre was added. Finally, questions about the cost of acquisition and repairs, and costs at own account (e.g., adjustments to the house, car, or for sports/hobbies) were added. Despite the fact that the health insurer reimburses most of those costs, prosthesis users do have insight into the costs of purchasing and repairing their prosthesis, since their health insurer informs them about these costs. These total costs were explicitly requested in the questionnaire. Respondents were asked to fill out the cost questionnaires considering the upper limb prosthesis they used most. The recall periods of the Productivity Cost Questionnaire and Medical Consumption Questionnaire were adjusted to a period of one year, since prosthesis users often visit their rehabilitation team only a few times a year. The official recall periods of respectively three months and four weeks were considered too short to capture a reliable picture of the costs related to upper limb prosthesis use. To value indirect costs related to productivity loss, the friction cost method was used with a friction period of 85 calendar days, including the value of unpaid work [20]. The most recent Dutch reference prices from 2014, which represent the average unit costs, were indexed with inflation rates retrieved from the Statistics Netherlands’ database [35]. The frequency of medical consumption, including doctor visits and hospital admissions was reported by the respondents. Subsequently, these were valued at Dutch standard prices. For a consultation with a hand therapist, who originally were all educated as occupational therapists or physiotherapists, the same cost as for a consultation with a physiotherapist was used. The average price for a consultation with the prosthetist and the technician was derived from expert opinion. Most of the reported medication costs were not related to upper limb prosthesis use, and therefore, we decided to exclude medication costs from our analyses. Absenteeism and presenteeism, defined as respectively the unscheduled absence and lost productivity caused by not fully functioning of an employee, were determined according to the Dutch cost guideline [36]. Because costs were determined over one year, discounting was not necessary.

### Data collection and analyses

Between May and July 2020 postal surveys were sent to adult prosthesis users (≥18 years) with acquired or congenital upper limb absence at or proximal of the wrist from two large orthopaedic workshops with multiple branches all over the country, and nine out of 10 Dutch rehabilitation centres that prescribe upper limb prostheses. If no response was received within 5–11 weeks, a reminder was sent. Participants who only completed the paper part of the survey, but not the digital part (i.e., PUF-ULP), were sent a request to complete the digital part of the survey as well. The latter was only possible if participants provided address information on the returned survey. Participants received an incentive of €10,- for completing the surveys.

RedCap data capture tools were used for data management [37,38]. The database was checked for duplicates, which was only possible if participants provided address information on the returned survey. The most complete survey was included for analyses. Additionally, participants with bilateral upper limb absence, aged under 18 years, with upper limb absence distal from the wrist, without upper limb absence, and non-prosthesis users were excluded from all analyses. Participants who only competed the PUF-ULP, but not the paper part of the survey (i.e., demographic data and EQ-5D) were excluded from the health economic evaluation, but were included in the estimation of the PUF-ULP weights, since the reliability of the estimation improves with a higher number of participants. The same applies for participants from whom the type of prosthesis could not be categorized based on the provided information (i.e., cosmetic/passive prostheses, body-powered prostheses, prostheses with tools/accessories, SHPs, MHPs).

### Health economic evaluation

#### Cost-effectiveness of SHP versus MHP

The incremental cost-utility ratio (ICUR) was calculated by dividing the difference in costs related to MHP and SHP use by the difference in effects measured with the EQ-5D-5L utility score. A quality-adjusted life year (QALY) describes the burden of a disease and includes both the quality and the quantity of life lived. One QALY reflects one year in perfect health. QALY scores range from 1 (perfect health) to 0 (dead). In the current study, a utility equals a QALY since prosthesis use does not affect life-years and the time-horizon is one year. In addition, an incremental cost-effectiveness ratio (ICER) was calculated with the difference in PUF-ULP score as the effect measure. Because the outcome of the PUF-ULP is not a score between 0 and 1, it was not feasible to express the effects as QALYs. The statistical uncertainty and robustness of results were estimated using bootstrapping. We used 5000 replications, simulating repetition of the study 5000 times with variations in the results regarding mean incremental costs and effects in each replication [39]. The results are presented graphically in a scatter plot showing the incremental costs of each simulation on the y-axis and the incremental effects on the x-axis. This cost-effectiveness plane (CE-plane) is divided into four quadrants: when simulations of the ICER/ICUR are delivered in the north-east quadrant (NE-Q), the MHP generates better health outcomes, but is also more expensive. The north-west quadrant (NW-Q) and south-west quadrant (SW-Q) are relevant when the MHP generates poorer health outcomes and/or lower costs. Results in the south-east quadrant (SE-Q) represent the MHP being definitively cost-effective compared to the SHP. The results of the bootstrap simulations were used to determine the probability of cost-effectiveness at various threshold values for willingness to pay (WTP) for a gain of one QALY. A WTP threshold of €20 000,- per QALY gained, is recommended for people with upper limb absence by The National Health Care Institute (NHCI) [40]. This was graphically presented in a cost-effectiveness acceptability curve (CEAC). This curve represents the probability that the MHP is cost-effective compared to the SHP, for a certain threshold value of the ICUR. A CE plane and CEAC were also constructed with PUF-ULP as the outcome.

#### Missing data

A significant part of the acquisition and repair costs appeared to be unknown by the participants. This missing data were, if possible, replaced by the estimations of repair costs from the expert opinions of the financial employees of two large orthopaedic workshops with several branches in the Netherlands (Table 1). They estimated the repair cost of the most common repairs per prothesis type. Only if a participant did not provide the acquisition or repair costs, these were replaced by the costs based on expert opinions. To account for missing data in the EQ-5D-5L and PUF-ULP in the sensitivity analyses, multiple imputation (MI) was applied. MI was performed based on predictive mean matching by using “mi impute pmm.” The following covariates were included in the MI model: hours of wearing the prosthesis per day, age, sex, type of prosthesis used most, years since prescription, and level of upper limb absence.

**Table 1.** Acquisition and repair costs that were applied when a participant did not provide values for those costs in the survey.

Prosthesis type	Acquisition cost	Repairs	Repair costs
MHP			
Below elbow	€40 700	New glove	€800
Above or through elbow	€47 350	New battery	€1600
		Repair finger/thumb	€1075
		General maintenance	€1000
		Other^a^	€1136
SHP			
Below elbow	€13 650	New glove	€460
Above or through elbow	€17 650	New battery	€1550
		New socket	€400
		Repair socket	€200
		New inner hand	€160
		Rewire new electrode	€210
		General maintenance	€100
		Other^a^	€566
CP			
Below elbow	€3600	New glove	€320
Above or through elbow	€6750	Inner hand replacement	€160
		Socket replacement	€500
		Alignment	€300
		Other^a^	€320
BP			
Below elbow	€4800	New glove	€460
Above or through elbow	€7050	New bandage	€235
		Inner hand replacement	€160
		New cable	€180
		Cosmetic repair	€400
		Repair hand	€350
		Other^a^	€296
Prostheses with tools/accessories			
Below elbow	€4300	Change cable	€60
Above or through elbow	€6300	Replacement of protection of the tool	€50
		Change hook	€400
		Change tool	€300
		Other^a^	€203

MHP: multi-grip myoelectric hand prosthesis; SHP: standard myoelectric hand prosthesis; CP: cosmetic/passive prosthesis; BP: body-powered prosthesis; Below elbow: transradial or wrist disarticulation; Above or through elbow: elbow disarticulation, transhumeral, shoulder disarticulation, and forequarter.

^a^ For the category “other,” the average of all reported repair costs by the orthopaedic workshop was depicted.

### Statistical analyses

Continuous variables were checked for normality and equality of variances with Q-Q plots, Kolmogorov-Smirnov test, and Levene’s test. Differences in demographic characteristics, the EQ-5D-5L utility and VAS scores, PUF-ULP scores, and costs between all groups of prosthesis users were evaluated using a Kruskal-Wallis test for continuous variables and a Pearson’s χ^2^ test for categorical variables. A Fisher’s exact test was carried out if sample sizes were too small for a Pearson’s χ^2^ test. All tests were performed two-tailed. Statistical significance was set at α < 0.05. Since none of the continuous variables met the assumptions of a one-way ANOVA, only Kruskal-Wallis tests were performed. Mann Whitney tests were used to follow up statistical differences. For the latter, a Bonferroni correction was applied. Therefore, all effects of the Mann-Whitney *post hoc* testing were reported at α < 0.005. Data analyses were performed with IBM SPSS statistics version 23 (IBM Corporation, Armonk, NY, USA) and Stata version17BE (StataCorp, College Station, TX, USA).

## Results

### Study population

**Figure 1.**
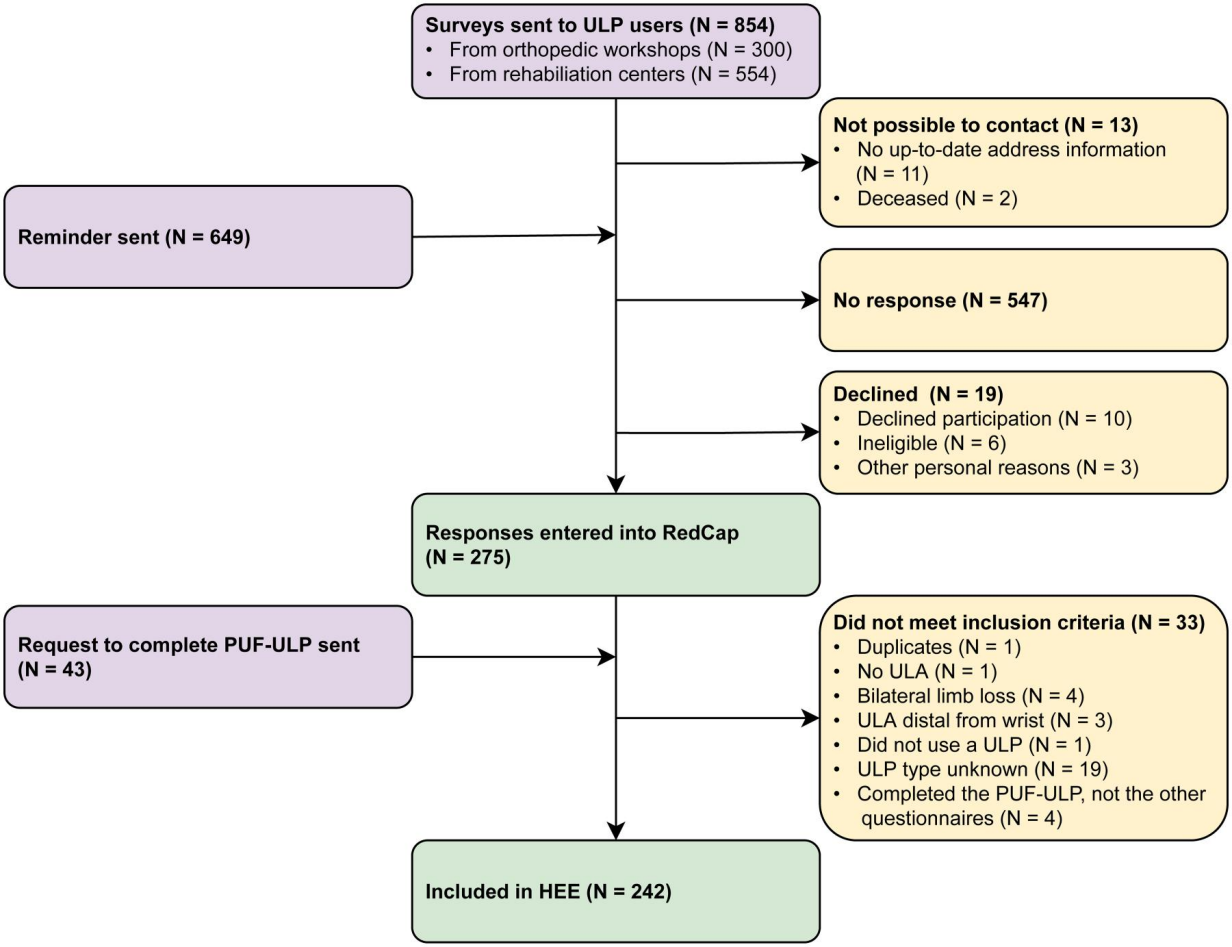
Flowchart of survey distribution (purple), response (yellow), and inclusion process (green) for the health economic evaluation (HEE). ULP: upper limb prosthesis; ULA: upper limb absence; PUF-ULP: patient-reported outcome measure to assess the preferred usage features of upper limb prostheses.

Surveys were sent to 854 participants, of which 275 surveys were returned (Figure 1). Ten respondents did not meet the inclusion criteria. Additionally, for 19 respondents the type of upper limb prosthesis was unknown, and four respondents only completed the digital PUF-ULP, which ultimately led to the inclusion in the health economic evaluation of 242 respondents (138 females, 104 males; mean age 57.6 ± 15.9 years; Table 2). MHP users were significantly younger compared to SHP users and people using a prosthesis with tools/accessories. Furthermore, we found a significant association between sex and type of prosthesis used. Based on the relative risk, women were twice as likely to own a CP as men. Conversely, men were respectively 2.3, 1.5, 1.7, and 1.2 times as likely as women to own an MHP, SHP, BP, or a prosthesis with tools/accessories. Additionally, we found a significant association between level of upper limb absence and type of prosthesis used. Based on the relative risk, people with upper limb absence above or through the elbow were respectively 1.6 and 2.3 as likely to use a CP or BP most. Conversely, people with an amputation below the elbow were respectively 2.7, 1.9, and 2.4 as likely to use an MHP, SHP, or a prosthesis with tools/accessories.

**Table 2.** Demographic characteristics of the 242 participants stratified by type of prosthesis.

	MHP (*n* = 28)	SHP (*n* = 78)	CP (*n* = 97)	BP (*n* = 26)	Prosthesis with tools/ accessories (*n* = 13)	Total (*n* = 242)	*p* value
Age, M ± SD, *n* = 239^a^	49 ± 14	59 ± 16	57 ± 17	61 ± 16	64 ± 10	58 ± 16	0.02*
Sex, *n* (%)							
Male	21 (75)	52 (67)	39 (40)	18 (69)	8 (62)	138 (57)	0.00*
Female	7 (25)	26 (33)	58 (60)	8 (31)	5 (38)	104 (43)	
Origin of ULA, *n* (%)							
Congenital	13 (46)	23 (29.5)	41 (42)	10 (38.5)	2 (15)	89 (37)	0.15
Acquired	15 (54)	55 (70.5)	56 (58)	16 (61.5)	11 (85)	153 (63)	
Side of ULA, *n* (%)							
Right	13 (46)	36 (46)	37 (38)	11 (46)	4 (31)	103(43)	0.51
Left	15 (54)	42 (54)	60 (62)	15 (54)	9 (69)	139 (57)	
Employed, *n* (%)^a^							
Yes	18 (64)	36 (46)	44 (45)	14 (54)	7 (54)	119 (49)	0.62
No	10 (36)	40 (51)	52 (54)	11 (42)	6 (46)	119 (49)	
Years since prescription^b^, M ± SD, *n* = 237^a^	3 ± 2	5 ± 9	6 ± 10	6 ± 10	7 ± 13	5 ± 9	0.74
Level of education, *n* (%)^a,c^							
Lower	7 (25)	21 (27)	34 (35)	7 (27)	4 (31)	73 (30)	0.69
Middle	10 (36)	29 (37)	28 (29)	6 (23)	6 (46)	79 (33)	
Higher	11 (39)	28 (36)	35 (36)	13 (50)	3 (23)	90 (37)	
Hours wearing the prothesis per day, M ± SD, *n* = 242	8 ± 6	11 ± 5	10 ± 6	10 ± 7	7 ± 7	10 ± 6	0.07
Level of ULA, *n* (%)^a^							
Below elbow	24 (86)	63 (81)	57 (59)	13 (50)	11 (85)	168 (69)	0.00*
Above or through elbow	4 (14)	15 (19)	40 (41)	13 (50)	2 (15)	74 (31)	
Number of prostheses,* n*(%)							
One	21 (75)	62 (80)	80 (83)	20 (77)	8 (62)	191 (79)	0.55
Two or more	7 (25)	16 (20)	16 (17)	6 (23)	5 (38)	50 (21)	

MHP: multi-grip myoelectric hand prosthesis; SHP: standard myoelectric hand prosthesis; CP: cosmetic/passive prosthesis; BP: body-powered prosthesis; M: mean; SD: standard deviation; *n*: number of participants; ULA: upper limb absence.

^a^ Some variables have missing responses and do therefore not add up to 100% or 242 participants.

^b^ Of current prosthesis.

^c^ Low: no education or lower vocational education; middle: middle vocational education; high: higher education such as university of applied sciences or university (BSc/MSc).

*Significant at α < 0.05.

### Costs

The acquisition costs between all types of prostheses differed significantly, except between the CP and the prostheses with tools/accessories (Table 3). Myoelectric prostheses, especially MHPs, were the most expensive prostheses compared to the other types. Repair costs were significantly higher for both the MHP and SHP groups compared to the CP group. Healthcare costs were significantly different between groups, however, *post hoc* testing showed no significant differences for the separate comparisons. The travel expenses of the MHP and SHP groups were significantly higher than of the CP group. Both MHP and SHP users had significantly more total costs than CP, BP, and prostheses with tools/accessories users. Finally, the MHP users incurred significantly more direct costs compared to SHP users (see Supplementary file 2 for disaggregated costs).

**Table 3.** Inflation indexed mean costs over a 12- month period (€) per patient depicted per type of prosthesis.

	MHP	SHP	CP	BP	Prosthesis with tools/ accessories	Total	*p* value
Acquisition	42 592 ± 10 871	14 288 ± 4392	4842 ± 2331	5798 ± 1949	4354 ± 686	12 331 ± 12 658	0.00*
Repair	2659 ± 7931	1249 ± 2287	302 ± 389	366 ± 347	296 ± 613	887 ± 3067	0.00*
Healthcare	2691 ± 4780	2344 ± 7934	1514 ± 5079	1393 ± 3291	2672 ± 4674	1967 ± 5951	0.05*
Travel	104 ± 153	85 ± 230	26 ± 44	67 ± 149	61 ± 90	60 ± 155	0.00*
Productivity losses	6066 ± 9910	5535 ± 9831	3447 ± 8628	3674 ± 8552	4595 ± 8669	4509 ± 9166	0.07
Total cost	54 112 ± 17 221	23 501 ± 14 362	10 132 ± 10 896	11 298 ± 9388	11 977 ± 10 210	19 754 ± 18 727	0.00*

Values are presented as mean ± SD.

MHP: multi-grip myoelectric hand prosthesis; SHP: standard myoelectric hand prosthesis; CP: cosmetic/passive prosthesis; BP: body-powered prosthesis.

*Significant at α < 0.05.

### Estimation of PUF-ULP weights

The estimation of PUF-ULP weights was based on 171 responses (Supplementary file 3). Respectively, 10 and 15 respondents completed the PUF-ULP multiple times or partially and were therefore excluded. However, after completion of the weight estimations, two additional respondents who completed the PUF-ULP only partially were identified. Since these two respondents completed only one or two comparisons with hypothetical prosthesis experiences in the second task, the effect on the final weight estimations was negligible and we decided not to rerun the estimations of the PUF-ULP weights. The two worst response levels for the items “user-friendly” and “reliability” were merged, as less than two participants rated their prosthesis experiences on these response levels. The applied weight estimations for the PUF-ULP are added in Supplementary file 4. Hence, rating the item “reliability” on the worst response level results in 0.18 points score decrease, while rating the items “work, hobby, and household,” “wearing comfort,” and “independence” in the worst response level results in respectively 2.19, 1.83, and 1.76 points decrease.

### Outcome measures

Both, the EQ-5D-5L utility and VAS scores, did not differ between people using different types of prostheses (Table 4). Furthermore, the EQ-5D-5L scores did also not differ between people using different types of prostheses.

**Table 4.** Comparison of the EQ-5D-5L and the patient-reported outcome measure to assess the preferred usage features of upper limb prostheses (PUF-ULP) scores by type of prosthesis used most.

	MHP	SHP	CP	BP	Prosthesis with tools/ accessories	Total	*p* value
EQ-5D-5L: utility score	0.80 ± 0.21	0.84 ± 0.17	0.86 ± 0.15	0.85 ± 0.15	0.72 ± 0.22	0.84 ± 0.17	0.21
	*n* = 26	*n* = 75	*n* = 90	*n* = 25	*n* = 12	*n* = 228^a^	
EQ-5D-5L: VAS score	77.5 ± 16.1	80.2 ± 17.0	79.0 ± 17.0	81.1 ± 14.7	77.3 ± 9.0	79.4 ± 16.3	0.42
	*n* = 28	*n* = 76	*n* = 94	*n* = 26	*n* = 13	*n* = 237^a^	
PUF-ULP	8.76 ± 2.45	8.93 ± 1.83	8.53 ± 2.13	9.13 ± 2.65	7.34 ± 2.34	8.71 ± 2.16	0.18
	*n* = 20	*n* = 57	*n* = 55	*n* = 19	*n* = 8	*n* = 159^a^	

Values are presented as mean ± SD.

MHP: multi-grip myoelectric hand prosthesis; SHP: standard myoelectric hand prosthesis; CP: cosmetic/passive prosthesis; BP: body-powered prosthesis; VAS: visual analogue scale; *n*: number of participants.

^a^ Due to missing responses, the total number of participants does not add up to 242.

### Cost-utility and cost-effectiveness analyses: MHP versus SHP

Before bootstrap replication, the difference in EQ-5D-5L utility scores between MHP and SHP users was −0.042 (95% CI: −0.040 to −0.124). The difference in estimated yearly costs was €30 611 (95% CI: €23 990 to €37 232), which yielded an ICUR of €−728 833 per QALY.

Additionally, the incremental difference between PUF-ULP scores of MHP and SHP was −0.163 (95% CI: −0.874 to 1.200), resulting in an ICER of €−187 798 per point gained on the PUF-ULP.

For the EQ-5D-5L utility scores and PUF-ULP scores respectively, five (4.72%) and 29 (27.36%) responses were missing among the MHP and SHP users. For the bootstrap replication, these missing values were imputed using MI prior to sensitivity analyses. After the bootstrap, the mean incremental cost was €30 568 (range: €18 916 to €45 075) between MHP and SHP users. The mean incremental QALYs was −0.041 (range:-0.198 to 0.093). The estimation of mean ICUR after bootstrap was influenced by extreme ICUR values caused by QALY differences close to zero (mean: €−2 652 200 per QALY, 95% CI: €−3 562 201 to €8 866 601) and was considered to be non-informative.

**Figure 2.**
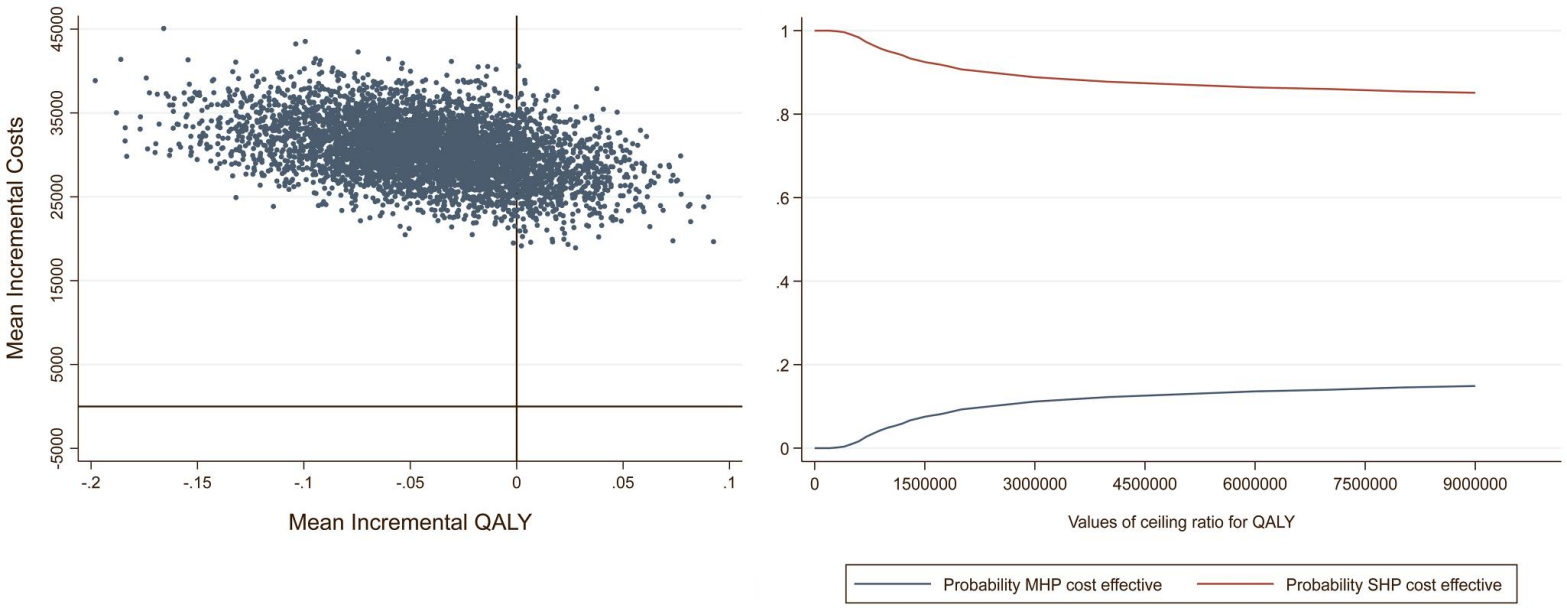
Left panel: cost-effectiveness plane (CE-plane) of the quality-adjusted life-years (QALY) analysis with the mean incremental QALYs depicted on the X-axis and the mean incremental costs (€) on the Y-axis. Right panel: cost-effectiveness acceptability curve (CEAC) of the QALY analysis with the Willingness-To-Pay (WTP) threshold depicted on the X-axis and the probability of the MHP and SHP being cost-effective on the Y-axis.

Concerning the CE-plane for the QALY analysis, the majority of the dots fell within the NW-Q indicating a lower utility and higher cost for the MHP in comparison with the SHP (Figure 2, left panel). Finally, the CEAC regarding the QALY analysis (Figure 2, right panel) displays that for all WTP thresholds the SHP has a higher probability of being cost-effective compared to the MHP.

**Figure 3.**
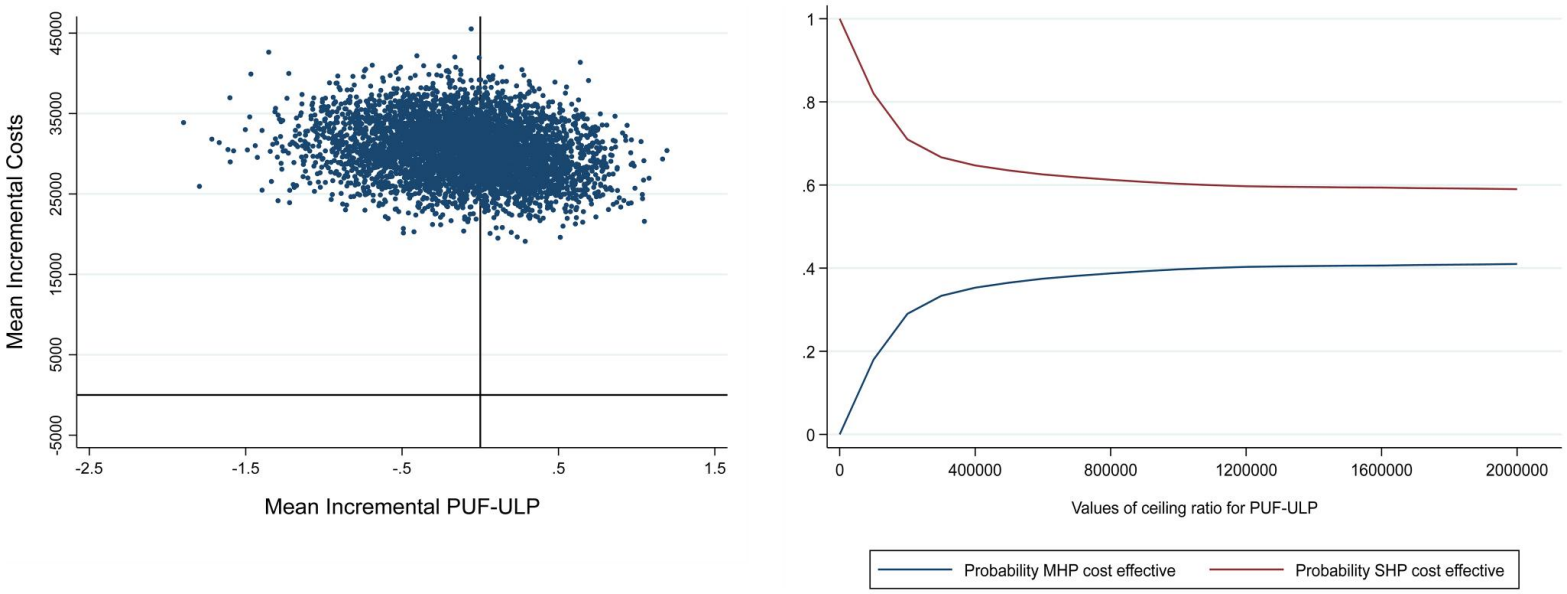
Left panel: cost-effectiveness plane (CE-plane) of the patient-reported outcome measure to assess the preferred usage features of upper limb prostheses (PUF-ULP) analysis with the mean difference in estimated PUF-ULP scores between the MHP and SHP on the X-axis and the mean difference in estimated costs on the Y-axis. Right panel: cost-effectiveness acceptability curve (CEAC) of the PUF-ULP analysis Willingness-To-Pay (WTP) threshold depicted on the X-axis and the probability of the MHP and SHP being cost-effective on the Y-axis.

The analysis with the PUF-ULP as outcome produced a mean incremental difference in costs of €30 449 (range: €19 109 to €45 525) in disadvantage of the MHP compared to the SHP and a mean incremental effect of −0.102 (range:-1.897 to 1.194). Similar to the ICUR, mean ICER after bootstrap was not informative due to small effect differences (mean: €−27 935 per point gained on the PUF-ULP, 95% CI: €−215 083 to €159 213). In the CE-plane (Figure 3, left panel), most dots fell within the NW-Q and fewer in the NE-Q indicating respectively a lower effect with higher cost and a higher effect with a higher cost for the MHP in comparison with the SHP. The CEAC displays that for WTP thresholds up to two million Euros the MHP has a lower probability of being cost-effective than the SHP, however, when the WTP threshold increases, the probability for the MHP to be cost-effective increases too.

## Discussion

In this nationwide survey study, including 242 upper limb prosthesis users, the costs, health-related quality of life, and user experiences associated with the use of different upper limb prosthesis types were investigated. Additionally, the cost-effectiveness of MHPs and SHPs were compared in more detail. To the authors’ knowledge, this is one of the first health economic evaluations about upper limb prostheses [10–12]. Results showed that (1) myoelectric hands were more expensive compared to CPs, BPs, and prostheses with tools/accessories; (2) within the group of myoelectric prosthesis MHPs were on average more than twice as expensive as SHPs; (3) the total cost of the CPs, BPs, and prostheses with tools/accessories varied little from each other; (4) the differences in the total cost between the prosthesis types were for the most part explained by the acquisition cost; (5) the health-related quality of life and user experiences, measured with the PUF-ULP, did not differ between people using different prosthesis types; (6) the ICER and ICUR results indicated that MHPs were more expensive and less effective compared to SHPs.

In the literature, mixed claims regarding health-related quality of life and prosthesis experiences of people using different prosthesis types were made. For instance, the survey study of Yamamoto et al. [3], that included 174 participants, found higher EQ-5D-5L utility scores in CP users compared to MHP/SHP and BP users. Šosterič et al. [41] found similar results, although they evaluated prosthesis satisfaction: both functional and overall prosthesis satisfaction was highest in people using a CP, followed by people using BPs and MHP/SHPs. However, three other studies, all from Resnik et al. found no differences in health-related quality of life [4,18] nor for prosthesis satisfaction [18,42]. The latter is in line with the results of the current study, in which we found no differences in health-related quality of life nor in the user experiences between different prosthesis types. However, it should be noted that, in contrast to the above-mentioned studies, the user experiences in our study were expressed to the extent a prosthesis meets the preferred usage features of upper limb prostheses, which implies another concept than prosthesis satisfaction. The finding that prostheses with more functional options, such as MHP/SHPs and BPs, do not result in higher health-related quality of life and better user experiences, may be surprising. However, multiple studies already suggested that the preferences of upper limb prosthesis users are generally consistent with the purpose of their prosthesis, in other words CP users often prioritize comfort and appearance, while MHP/SHP users often prioritize function [22,43,44]. As a consequence, CPs or prostheses with tools/accessories can meet the needs of an individual equally well, or sometimes maybe even better.

Results indicated that MHPs were not cost-effective compared to SHPs. The CEACs (Figures 2 and 3) show that the lines representing the SHPs and MHPs are probably not going to cross each other at all. Thus, even with a very high WTP threshold, MHPs will probably not result in health benefit or better user experiences compared to SHPs. However, from a technical perspective, the MHP has advantages over the SHP. We may therefore wonder why these advantages are not yet apparent in practice. One reason might be that the MHP users are not skilled enough to control their prosthesis optimally. Previous studies indicated that prosthesis training is of utmost importance to improve benefits of especially MHPs [4,45]. Future studies should therefore include more information about the prosthetic skills and training of the participants included. Another reason might be the difficulties of switching grips with the MHPs. Generally, both MHPs and SHPS are controlled by two electrodes placed on the skin above flexors and extensor muscles of the wrist or elbow. To switch grips when using an MHP, trigger signals generated by the muscles, such as co-contraction or double pulses, have to be made [8,46]. Switching grips is experienced by the MHP-users as non-intuitive, cognitively demanding, and slow [47]. Future studies should investigate the potential of, e.g., pattern recognition control to overcome this problem [47]. Furthermore, other disadvantages of MHPs regarding the robustness, durability, appearance, noise, and comfort were acknowledged in literature [48], which may also explain the results of the current study.

We used a new preference-based outcome measure that was specifically developed for people with upper limb absence, the PUF-ULP [22]. The weights for the response levels of each item can be summed, which enabled us to express the match between the user and their prosthesis with a single score. Interestingly, the items “work, hobby, and household,” “wearing comfort,” and “independence” had a relatively high impact on the PUF-ULP scores compared to other items, while “reliability” had a relatively small impact (Supplementary file 4). A downside of using a new outcome measure is that direct comparisons with existing literature are not possible. Using an outcome measure that has been used more often, such as one of the variants of the Trinity Amputation and Prosthesis Experience Scale (TAPES) [49,50] or the Orthotics and Prosthetics Users’ Survey (OPUS) [51,52], would solve this problem. However, these outcome measures were developed with, respectively, a primary focus on the lower limb [49] or a focus on orthotics and prosthetics from both lower and upper limbs [51]. In contrast, the contents of the PUF-ULP were developed specifically for people with upper limb absence [22]. To overcome the aforementioned limitations related to the PUF-ULP, we also used the EQ-5D-5L as a generic outcome measure for the health economic evaluations, to enable comparisons with the literature.

Since the MHP cannot be considered cost-effective compared to the SHP, we advise being careful with prescribing MHPs. However, groups of MHP and SHP users were compared, not individuals. Possibly, the MHP offers benefits for specific users within the entire group of prosthesis users. Therefore, it is important, when considering prescribing an MHP in clinical practice, to carefully test the advantages of the MHP for each individual compared to the functionality the SHPs offer. Additionally, clinicians can use the information about cost-effectiveness of the MHP for their patient education to increase the patients’ awareness about acquisition costs, which may stimulate patients to consider other options more seriously. To further improve prosthesis prescription procedures, future research should include a larger group of MHP-users to investigate for whom the MHP does yield benefits in terms of health-related quality of life and user experiences and for whom not.

Some limitations should be mentioned. First, we did not have information on non-respondents. Second, we could not calculate a response rate because participants from both rehabilitation centres and orthotic workshops were sent a survey and as a consequence, many participants probably received more than one survey. Although we noted on the title page of the survey that the participants should only fill out the survey once, we might not have identified all duplicates, since those could only be checked if participants provided personal details. The advantage of inviting participants from both sources was that a larger sample was reached in this way. Third, we asked participants to answer the questions regarding the upper limb prosthesis they used most in the survey, while 21% were in possession of multiple prostheses. Fourth, while the PUF-ULP was meant as a practical and easy to use tool, 69 participants did not fill out the PUF-ULP, 15 only partially, and 10 multiple times. Possibly, the assignment was not clear enough or filling out a survey digitally was difficult for some people. However, the second task of the PUF-ULP is dependent on the provided responses to the first task. Therefore, a paper version of the PUF-ULP was not feasible. Fifth, the weights for each response level of the nine included items were estimated based on the responses of 171 individuals (Supplementary file 3). This is a modest sample that is producing estimated weights that are not very precise. However, an advantage of the used measurement framework is that the estimated weights will become more precise over time if more people use the PUF-ULP. Sixth, the generalizability of the current study is limited due to differences in the cost of resource usage between different countries. Seventh, participants were often not conscious about the acquisition and repair cost of their upper limb prosthesis, which was addressed by replacing those missing values by the prices of acquisition and repairs according to expert opinions. Eighth, the recall periods of the Productivity Cost Questionnaire and Medical Consumption Questionnaire were prolonged to one year to capture a complete picture of prosthesis-related cost, but this also may have led to an increase in the recall bias. However, a time horizon of one year is still relatively short for cost-effectiveness studies carrying out a modelling approach. Due to several methodological issues, a modelling approach would, however, lose much of its impact in the current study. Ninth, participants were asked to provide the costs related to upper limb prosthesis use in the most recent year and not their first year after prescription of their upper limb prosthesis. This was done to reduce the already prolonged recall bias. This may have led to an underestimation of costs, since cost are probably higher in the first year after upper limb prosthesis prescription due to prosthesis training and appointments with the prosthetist for refinements. The best solution to overcome the last two limitations would be a prospective cost-effectiveness study regarding upper limb prostheses. However, the small Dutch population of people with upper limb absence complicates such a design. Considering the different health care systems in different countries, an international study could be complicated as well, but may offer a solution. Another option may be a prospective study spanning over a long period of time.

To conclude, this study took the first step to reveal the cost-effectiveness of upper limb prosthesis related healthcare. Results indicated that myoelectric prostheses, especially the MHPs, were most expensive compared to other types of upper limb prostheses, while no relevant differences in health-related quality of life and user experiences were apparent. Furthermore, the MHP was not cost-effective compared to the SHP. However, the latter finding was based on group means, and it might be that individual patients can benefit from an MHP. Future studies should therefore investigate for which specific individuals the MHP may be effective and how the disadvantages of MHPs can be surmounted. The information gathered in this study can be used to educate professionals and patients about the costs and effects of upper limb prostheses to increase awareness and involvement in the prosthesis selection process. Furthermore, when considering prescribing an MHP, careful evaluation and assessment of the potential advantages for the future user of an MHP over the SHP are recommended to prevent prescribing an unnecessary expensive upper limb prosthesis.

## Data Availability

Data that support the findings of this study are available on DataVerseNL: https://doi.org/10.34894/TVITPL
